# Establishment of an Autoimmune Premature Ovarian Insufficiency Mouse Model with Proteomic Analyses: An Exploratory Study

**DOI:** 10.3390/ijms27104270

**Published:** 2026-05-11

**Authors:** Ying Tian, Jiaqi Zhou, Xinyi Pei, Feiran Liu, Feiyang Diao

**Affiliations:** 1 The Center for Clinical Reproductive Medicine, State Key Laboratory of Reproductive Medicine and Offspring Health, The First Affiliated Hospital of Nanjing Medical University, Nanjing Medical University, Nanjing 212028, China; tianying@stu.njmu.edu.cn (Y.T.);; 2 State Key Laboratory of Reproductive Medicine and Offspring Health, Nanjing Medical University, Nanjing 211166, China; jq2000@stu.njmu.edu.cn (J.Z.);

**Keywords:** premature ovarian failure, autoimmunity, mouse model, proteomics, immune imbalance

## Abstract

Premature ovarian insufficiency (POI) impairs fertility and health in reproductive-age women, with autoimmune factors contributing to 4–30% of cases. To investigate immune dysregulation in POI, we developed two mouse models using pZP3 induction: regular immune (RE-POI) and enhanced immune (EN-POI) cycles. The EN-POI model exhibited stable, irreversible ovarian dysfunction, including disrupted estrous cycles, hormonal changes (elevated FSH, decreased AMH, and estradiol), follicular depletion, and infertility. Immune profiling demonstrated consistent T-lymphocyte imbalance across both RE-POI and EN-POI model groups, characterized by expanded splenic CD4^+^ T cells, diminished regulatory T cells, elevated systemic inflammatory cytokines, and ovarian fibrosis. Proteomic comparison between the control and EN-POI groups identified 198 differentially expressed proteins, mainly enriched in immune and inflammatory pathways. Based on these differential proteins, subsequent network analysis further identified six key hub proteins, namely Mmp9, Isg15, Ikbke, Siglec1, Pf4, and Cdkn1b. This study establishes a stable autoimmune POI model, elucidates T-cell imbalance with cytokine storm and fibrosis, and identifies key molecules linking immune abnormalities to ovarian failure, offering new insights into POI research.

## 1. Introduction

POI is a clinical syndrome characterized by ovarian failure before the age of 40, presenting with amenorrhea, elevated gonadotropin levels, and decreased estrogen levels. It significantly impacts women’s fertility and long-term health, and it increases the risk of cardiovascular disease and osteoporosis [1,2,3]. The etiology of this condition is complex and multifaceted, with autoimmune factors considered to be one of the causes of POI [4].

Studies have shown that up to 30% of POI cases are associated with autoimmune abnormalities; autoantibodies against ovarian tissue-specific antigens are often detected in patients [5], accompanied by lymphocytic infiltration within the ovaries, suggesting that autoimmune attacks play a key role in the process of follicular depletion [6]. Therefore, a thorough understanding of the pathogenesis of autoimmune POI is crucial for the development of effective diagnostic and therapeutic strategies.

Although the clinical significance of autoimmune POI is widely recognized, the specific immune regulatory networks and the core molecular mechanisms underlying irreversible follicular damage remain unclear. Current research primarily relies on animal models to simulate disease progression, with POI models induced by autoantigens such as ZP3 being widely used [7,8,9]. However, existing models lack uniform standards and exhibit significant shortcomings in terms of stability and reproducibility of pathological features. Furthermore, most studies have only focused on a single type of immune cell or an isolated signaling pathway. Systematic analyses from a systems biology perspective are still lacking, including those on the ovarian local immune microenvironment, global proteomic changes, and their causal relationship with follicular failure. Therefore, this field faces an urgent, unresolved issue: developing stable animal models that closely mimic the natural history of human disease and combining omics technologies to systematically screen key molecular hubs and pathways between immune dysregulation and ovarian dysfunction.

This study established an autoimmune POI mouse model characterized by greater phenotypic stability and reduced spontaneous recovery, and comprehensively evaluated the estrous cycle, serum hormone levels, ovarian tissue morphology, follicle counts at various stages, and fertility testing. In terms of mechanistic exploration, we used flow cytometry to analyze changes in the proportions of immune cell subsets in the spleen, performed enzyme-linked immunosorbent assays (ELISAs) to measure serum levels of key inflammatory factors, and assessed the degree of ovarian fibrosis using Masson staining. In addition, by combining proteomic sequencing technology with the ESTIMATE algorithm, we performed bioinformatic scoring of the immune and stromal microenvironments in ovarian tissue. Finally, we constructed protein–protein interaction (PPI) networks and performed core module analysis of differentially expressed proteins to identify potential hub proteins, and conducted preliminary validation of clinical relevance using public databases.

This study provides new experimental evidence to elucidate the complex pathogenesis of autoimmune POI and lays the theoretical foundation for the future development of diagnostic biomarkers and therapeutic strategies based on immune modulation or targeting key molecules.

## 2. Results

### 2.1. Establishment and Validation of Immune-Mediated POI Mouse Models

**Figure 1:**
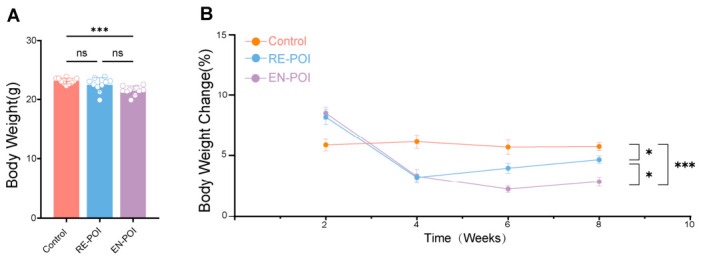
Body weight changes and statistical analysis of mice in each group. (**A**) Body weight statistics for mice in each group at the end of the experiment, n = 10. (**B**) Body weight change curves for mice over the experimental period, n = 10. * *p* < 0.05, *** *p* < 0.001; ns, no statistical difference.

To confirm the establishment of the POI model, we assessed the body weight of mice in each group (Control, n = 10; RE-POI, n = 10; EN-POI, n = 10) at fixed intervals every two weeks and plotted the body weight change curves (Figure 1A,B). The results showed that following the initial injection, mice in both the RE-POI and EN-POI groups exhibited a rapid, stress-induced increase in body weight. Interestingly, during the subsequent observation period, compared to the control group, which showed steady weight gain, the rate of weight gain in the RE-POI (*p* = 0.03) and EN-POI (*p* < 0.001) groups slowed significantly, with this effect being more pronounced in the EN-POI group (*p* = 0.04).

**Figure 2:**
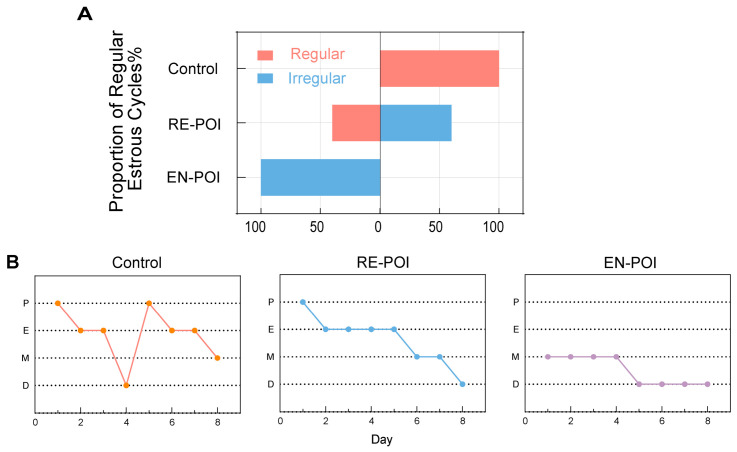
Estrous cycle status and representative cytological images (**A**) Proportion of mice with regular vs. irregular estrous cycles in each group, n = 10. (**B**) Representative images of the estrous cycle in mice from each group, n = 10.

After modeling, we collected vaginal lavage cell smears from mice in each group at the same time every day for 8 consecutive days to assess their estrous cycles (Figure S1). The normal estrous cycle in mice lasts approximately 4–6 days: the proestrus phase lasts 10 h, the estrus phase lasts 42 h, the diestrus phase lasts 12 h, and the metestrus phase lasts 48–72 h. We observed that, compared to the control group, both the RE-POI and EN-POI groups exhibited “menstrual irregularities” similar to those observed in human patients with POI, such as irregular cycles, reversed cycle sequences, or abnormal delays in specific estrus phases. Notably, the incidence of irregular estrous cycles was significantly higher in the EN-POI group (100%) than in the RE-POI group (60%) (Figure 2A,B).

**Figure 3:**
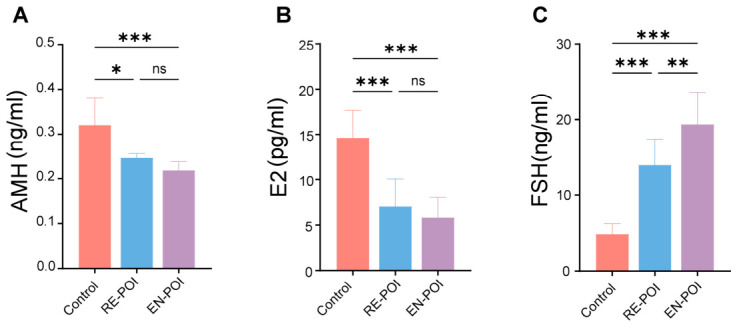
Serum levels of reproductive hormones in mice. (**A**) Serum AMH concentration; (**B**) serum E2 concentration; (**C**) serum FSH concentration, n = 10. * *p* < 0.05, ** *p* < 0.01, *** *p* < 0.001; ns, no significant difference.

We collected mouse serum and measured sex hormone levels using ELISA (Figure 3A–C). The results showed that follicle-stimulating hormone (FSH) levels in the serum of mice in the RE-POI (*p* < 0.001) and EN-POI (*p* < 0.001) groups were significantly higher than those in the control group, while estradiol (E2) (RE-POI, *p* < 0.001; EN-POI, *p* < 0.001) and anti-Müllerian hormone (AMH) (RE-POI, *p* = 0.05; EN-POI, *p* < 0.001) levels were significantly lower than those in the control group. Furthermore, there was a significant difference in FSH levels between the RE-POI and EN-POI groups (*p* = 0.003). This indicates that following ZP3 induction, mice develop a pathological phenotype of premature ovarian insufficiency, and that as the drug-induced cycle is prolonged, the pathological damage becomes more pronounced and stable.

### 2.2. Exacerbated Follicular Developmental Damage in EN-POI Mice

**Figure 4:**
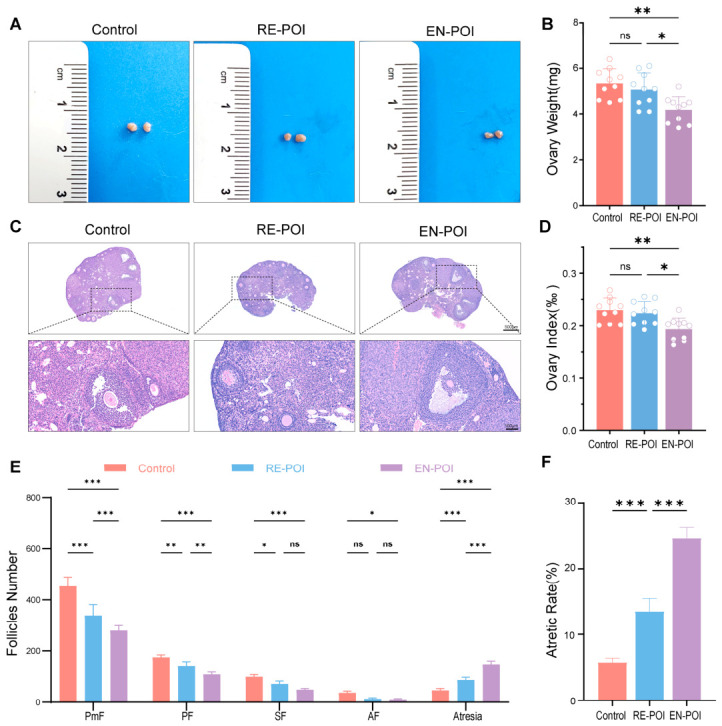
Ovarian morphological characteristics and follicular development in each group. (**A**) Representative macroscopic images of the ovaries in each group. (**B**,**D**) Statistics on ovarian weight and ovarian index in each group, n = 10. (**C**) Representative H&E-stained sections of ovarian tissue (**top**) and high-magnification views (**bottom**). (**E**) Statistics on the number of follicles at each stage: primordial follicles (PmF), primary follicles (PF), secondary follicles (SF), antral follicles (AF), and atretic follicles, n = 5. (**F**) Statistics on follicular atresia rates in each group, n = 5. * *p* < 0.05, ** *p* < 0.01, *** *p* < 0.001; ns, no statistical difference.

To further determine exactly what changes occurred in the mice, we collected ovaries from each group (Control, n = 5; RE-POI, n = 5; EN-POI, n = 5) and examined their histological morphology. The results revealed that ovarian volume, ovarian weight (*p* = 0.002), and ovarian index (*p* = 0.003) were significantly reduced in the EN-POI group relative to the control group. In contrast, no remarkable differences were found between the RE-POI group and the control group in ovarian weight (*p* = 0.74) and ovarian index (*p* = 0.91). Moreover, significant differences were observed between the RE-POI and EN-POI groups in both ovarian weight (*p* = 0.02) and ovarian index (*p* = 0.02) (Figure 4A,B,D). We also observed and counted the number of follicles at different developmental stages in the ovaries of mice from each group (Figure 4C,E). Compared with the control group, the counts of primordial follicles (PmF, *p* < 0.001), primary follicles (PF, *p* = 0.004), and secondary follicles (SF, *p* = 0.02) were markedly decreased in the RE-POI group, while all developing follicle subtypes were significantly reduced in the EN-POI group (both *p* < 0.001). Significant differences were also detected between the RE-POI and EN-POI groups in primordial (*p* < 0.001) and primary follicles (*p* = 0.005), yet no statistical distinction was found in secondary follicles (*p* = 0.08). In addition, the number of atretic follicles (AF) showed no obvious difference between the RE-POI and control groups (*p* = 0.06) but was notably elevated in the EN-POI group relative to controls (*p* = 0.03). Furthermore, the incidence of follicular atresia (Figure 4F) was significantly higher in both model groups than in the control group (both *p* < 0.001), and a remarkable difference also existed between RE-POI and EN-POI groups (*p* < 0.001).

### 2.3. Aggravated Reproductive Fertility Dysfunction in EN-POI Mice

**Figure 5:**
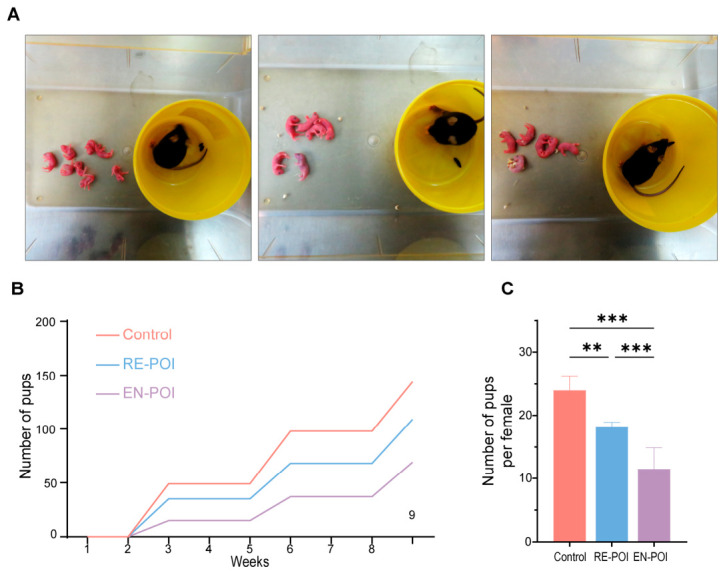
Fertility outcomes and litter size statistics. (**A**) Representative images of pups born to female mice in each group. (**B**) Cumulative number of pups born to female mice in each group across three rounds of fertility experiments, n = 5. (**C**) Statistics on the average number of pups per female in each group, n = 5. ** *p* < 0.01, *** *p* < 0.001.

A total of 5 mice per group were included in three consecutive rounds of fertility assessments (Figure 5A–C). The average number of pups per litter in the RE-POI (*p* = 0.002) and EN-POI (*p* < 0.001) groups was significantly lower than that in the control group, and the total number of offspring was also markedly reduced in both model groups. A significant difference was also detected between the RE-POI and EN-POI groups (*p* < 0.001). Interestingly, we observed that after two breeding cycles, the fertility of mice in the RE-POI group exhibited signs of recovery during the third breeding cycle.

### 2.4. Proteomics Reveals That Immune Dysregulation Is the Core Pathogenic Mechanism of POI

**Figure 6:**
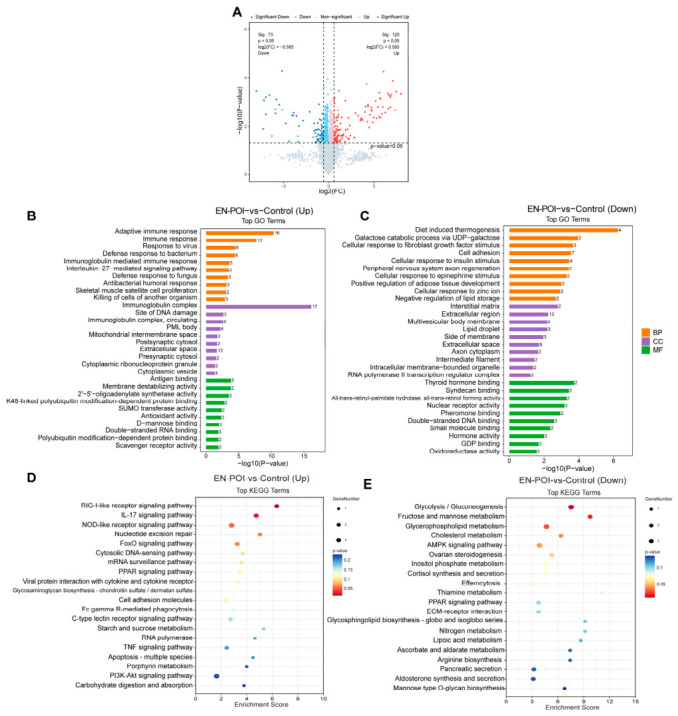
Proteomic sequencing and functional enrichment analysis of ovaries from POI and control mice. (**A**) Volcano plot of differentially expressed genes (DEPs) between the POI and control groups. (**B**,**C**) Top 20 GO functional enrichment analysis of upregulated and downregulated DEPs; bar length represents-log10 (*p*-value). (**D**,**E**) Top 20 KEGG pathway enrichment analysis of upregulated and downregulated DEPs.

To characterize proteomic alterations in ovarian tissue of autoimmune premature ovarian insufficiency mice, we performed liquid chromatography-tandem mass spectrometry (LC-MS/MS) analysis (Table 1, Table 2 and Table 3) on ovarian proteomes from the Control and EN-POI groups (n = 3, per group). We screened for 198 differentially expressed proteins (DEPs) using the criteria of fold change (FC) ≥ 1.5 or FC ≤ 1/1.5 and a *p*-value < 0.05, of which 73 were downregulated and 125 were upregulated. To more conveniently and intuitively display the distribution of proteins showing significant differences between the POI group and the control group, we constructed volcano plots based on the two variables of fold change and *p*-value (Figure 6A).

GO functional enrichment analysis indicated that upregulated DEPs were predominantly enriched in biological processes (BP) involving adaptive immunity, antiviral defense, and immunoglobulin-mediated immune responses, as well as cellular components (CC) (immunoglobulin complexes, extracellular domains) and molecular functions (MF) (antigen binding and polyubiquitination-dependent protein binding) (Figure 6B). Conversely, downregulated DEPs were mainly associated with biological processes of diet-induced thermogenesis, cellular response to fibroblast growth factors, and insulin receptor signaling, together with cellular components including extracellular matrix and intermediate filaments, and molecular functions such as RNA polymerase II transcription factor binding and hormonal activity (Figure 6C).

KEGG pathway enrichment analysis further revealed that upregulated DEPs were significantly enriched in the RIG-I-like receptor signaling pathway, IL-17 signaling pathway, and NOD-like receptor signaling pathway. These pathways are closely associated with innate immune activation and inflammatory responses (Figure 6D). In contrast, downregulated DEPs were enriched in pathways including glycolysis/gluconeogenesis, steroid biosynthesis, and ovarian steroid production, suggesting disrupted metabolic and hormonal homeostasis in the ovaries of POI mice (Figure 6E). Protein domain enrichment analysis showed significant enrichment of immunoglobulin-related domains, indicating that immune recognition and response regulation play a central role in the pathogenesis of POI (Figure S2B).

### 2.5. T Lymphocytes as the Primary Source of Immune Dysfunction in Immune-Mediated POI

**Figure 7:**
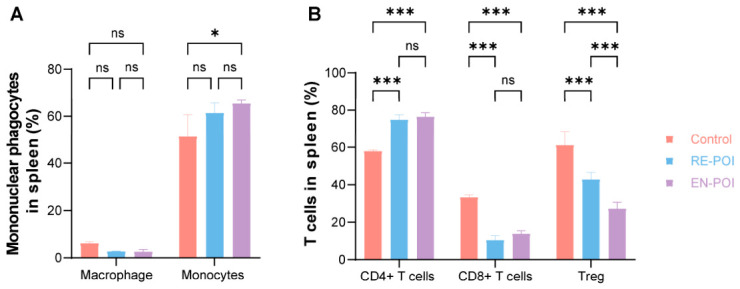
T lymphocytes as the primary source of immune dysfunction in immune-mediated POI. (**A**) Statistical analysis of the proportion of mononuclear phagocytes in the spleen of POI mice. (**B**) Statistical analysis of T cell subsets in the spleen of POI mice. * *p* < 0.05; *** *p* < 0.001; ns, no statistical difference.

To further investigate immune dysregulation in POI, we examined various immune cell subsets in the spleens of mice using flow cytometry, with n = 3 mice per group. The results showed no significant differences in the proportion of NK cells among the groups (Figure S3A,C). The proportion of B cells was reduced in the disease group compared to the control group, but there was no statistical difference between the RE-POI and EN-POI groups (Figure S3B,D). Among mononuclear phagocytes, no significant intergroup differences in macrophage proportions were detected (Control vs. RE-POI: *p* = 0.60; Control vs. EN-POI: *p* = 0.57; RE-POI vs. EN-POI: *p* > 0.99) (Figure 7A and Figure S4A). By contrast, monocyte proportions were significantly elevated in both model groups compared with the control group (Control vs. RE-POI: *p* = 0.03; Control vs. EN-POI: *p* = 0.004), whereas no remarkable difference existed between the RE-POI and EN-POI groups (*p* = 0.50).

Regarding T lymphocytes (Figure 7B and Figure S4B,C), compared with the control group, the proportion of CD4^+^ T cells was significantly higher (Control vs. RE-POI: *p* < 0.001; Control vs. EN-POI: *p* < 0.001) and the proportion of CD8^+^ T cells was significantly lower (Control vs. RE-POI: *p* < 0.001; Control vs. EN-POI: *p* < 0.001) in both RE-POI and EN-POI groups. Meanwhile, the proportion of Tregs was markedly lower in the RE-POI and EN-POI groups than in the control group (both *p <* 0.001), and a significant difference in Treg levels was also observed between the RE-POI and EN-POI groups (*p <* 0.001). These results indicate that immune dysfunction in the body during immune-mediated POI primarily originates from T lymphocytes.

### 2.6. Ovarian Fibrosis in POI Mice

**Figure 8:**
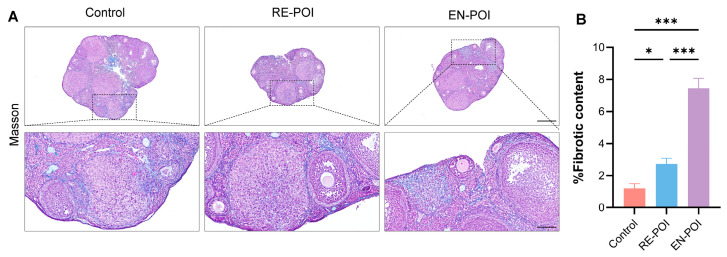
Ovarian fibrosis in POI mice. (**A**) Representative images of Masson’s trichrome staining of ovarian tissue; blue indicates collagen deposition. Scale bars: 250 μm (**top**), 100 μm (**bottom**). (**B**) Statistical analysis of the percentage of ovarian fibrosis. * *p* < 0.05; *** *p* < 0.001.

Fibrosis is one of the common pathological features of POI [10]. In this study, we assessed the ovarian fibrosis degree in mice of each group (n = 3) by Masson’s staining (Figure 8A,B). The results revealed that ovarian fibrosis levels were markedly elevated in both the RE-POI (*p* = 0.01) and EN-POI (*p* < 0.001) groups relative to the control group. Moreover, the fibrosis severity in the EN-POI group was significantly higher than that in the RE-POI group (*p* < 0.001), presenting the most severe fibrotic changes.

### 2.7. Cytokine Storm in POI Mice

**Figure 9:**
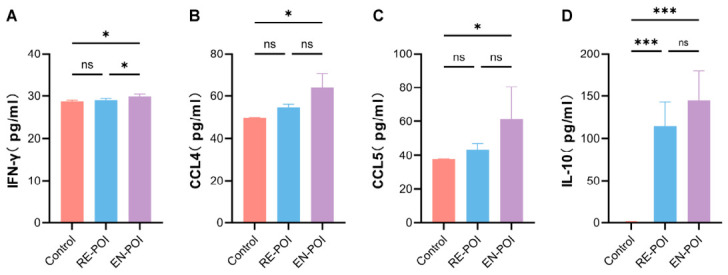
Cytokine storm in POI mice. (**A**–**D**) Multiplex flow cytometric analysis of serum IFN-γ, CCL4, CCL5, and IL-10 concentrations. * *p* < 0.05; *** *p* < 0.001; ns, no statistical difference.

We also performed multi-parameter flow cytometry to detect T cell-associated inflammatory cytokines in mouse serum, with n = 5 mice per group. The results revealed that the pro-inflammatory factor IFN-γ was significantly increased in the EN-POI group relative to the control group (*p* = 0.01), and its level was also markedly higher than that in the RE-POI group (*p* = 0.03), while no significant difference was observed between the RE-POI and control groups (*p* = 0.71) (Figure 9A). Regarding chemokines, CCL4 levels were significantly elevated in the EN-POI group compared with controls (*p* = 0.01), whereas no remarkable differences were found between the RE-POI group and the control group (*p* = 0.35) (Figure 9B). Similarly, CCL5 was significantly upregulated in the EN-POI group relative to the control group (*p* = 0.03), with no statistical distinctions detected in the RE-POI group versus controls (*p* = 0.69) (Figure 9C). For anti-inflammatory cytokines, IL-10 levels were significantly higher in both the RE-POI (*p* < 0.001) and EN-POI (*p* < 0.001) groups compared with the control group (Figure 9D).

### 2.8. Identification and Characterization of Key Hub Proteins That Regulate the Immune Response

**Figure 10:**
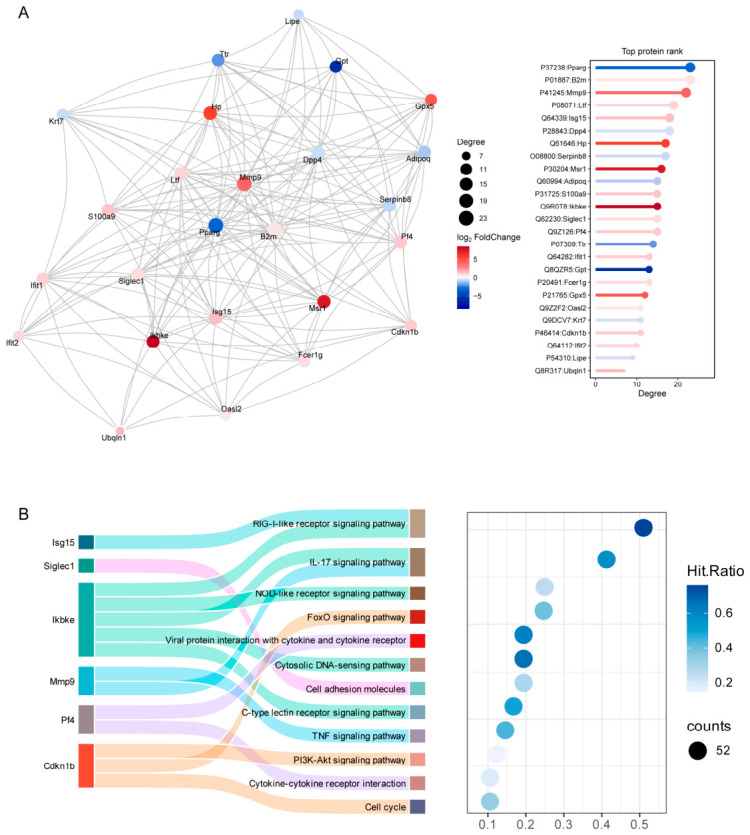
Protein–protein interaction (PPI) network and functional enrichment analysis of key differentially expressed proteins. (**A**) PPI network of core proteins. (**B**) Sankey diagram showing the relationship between core proteins and enriched signaling pathways.

To screen core regulatory molecules underlying immune-mediated ovarian dysfunction, we constructed a protein–protein interaction (PPI) network based on the top 25 differentially expressed proteins ranked by interaction degree. Combined with KEGG pathway enrichment analysis, six central hub proteins were identified, including Mmp9, Isg15, Ikbke, Siglec1, Pf4, and Cdkn1b. These hub molecules are located at the core of the immune regulatory network and are potential crucial mediators connecting immune dysregulation with follicular insufficiency (Figure 10A,B).

Additionally, we further validated the expression relevance of these hub genes using a public human POI follicular fluid RNA-seq dataset (GSE201276). The expression levels of Mmp9, Isg15, Ikbke, Siglec1, and Pf4 exhibited a negative correlation with the ovarian reserve marker AMH. Nevertheless, such correlation trends failed to reach statistical significance, which was mainly attributed to the limited sample size in the public database (Figure S5).

## 3. Discussion

Premature ovarian insufficiency is an endocrine disorder that significantly impacts the reproductive health and long-term quality of life of women of childbearing age. It is characterized by a decline in ovarian function before the age of 40, with clinical manifestations including amenorrhea and elevated gonadotropin levels [1]. Among the many causes, autoimmune factors are considered one of the key mechanisms underlying the development of POI [4,11]. Nevertheless, the exact pathogenesis remains unclear and may involve mechanisms such as a diminished oocyte pool, accelerated follicular atresia, or impaired folliculogenesis. In clinical practice, there are currently no reliable diagnostic markers or treatment methods for immune-mediated POI [12].

An ideal animal model serves as the cornerstone for in-depth elucidation of disease mechanisms and for the screening and evaluation of therapeutic interventions. Nevertheless, prominent biases and deficiencies persist in the establishment of animal models for POI research. At present, most studies heavily rely on chemotherapy-induced ovarian injury models. Although such models can recapitulate iatrogenic ovarian dysfunction, their underlying pathological mechanisms differ fundamentally from clinically prevalent autoimmune and idiopathic POI. On the other hand, currently available mouse models for immune-mediated POI lack unified methodological frameworks and standardized evaluation criteria [13]. In 1992, RHIM et al. reported that the mouse and human pZP3 proteins share 67% homology. pZP3 induces the production of AZP3Ab in mice; when this antibody binds to ovarian pZP3, it triggers an immune response that can cause symptoms similar to those of premature ovarian failure in humans, such as ovarian atrophy and anovulation [14]. In 1994, Smith and Hosid reported that anti-zona pellucida antibodies are associated with the onset of premature ovarian failure, confirming that these antibodies cause premature ovarian failure by accelerating the destruction and depletion of oocytes [15]. Subsequently, several other researchers used pZP3 to establish a model of immune-mediated premature ovarian failure [16]. Although previous studies have all employed the pZP3 model, there are significant discrepancies regarding the time required to establish the model and the criteria used for evaluation. There are notable shortcomings in terms of the stability of the animal model, the accuracy of pathological process simulation, and the analysis of systemic mechanisms, which limit our ability to gain a deeper understanding of the nature of the disease and to develop subsequent intervention strategies [13]. Therefore, developing animal models that more closely reflect the natural history of human disease and exhibit stable phenotypes, and elucidating at the systems level how immune dysregulation drives ovarian dysfunction, are critical scientific challenges that urgently need to be addressed in this field.

By optimizing the induction cycle of pZP3, this study compared the differences in fertility [17], hormone levels [18], and general condition [19] between conventional and enhanced immunization models of POI in mice, resulting in an immunological POI model that exhibits phenotypes similar to those reported in previous studies but with greater stability. The superiority of this model lies in its capacity to sustainably activate effector T cells and chronically suppress regulatory T cell (Treg) functions. This consequently disrupts systemic immune tolerance homeostasis and triggers an irreversible process of ovarian injury, accompanied by markedly impaired fertility without spontaneous functional recovery. According to clinical reports, the early symptoms of POI are relatively subtle; by the time most patients seek medical attention, their ovarian reserve has already been depleted, and the window for intervention has been missed [20]. This finding is particularly valuable for research. It also provides further clues for identifying key immune checkpoints in the progression of POI.

In addition, this study systematically compared the pathological features and molecular profiles of the disease models using multidimensional phenotypic analysis, quantitative proteomics, and immunological techniques. We found that the enhanced immunization regimen induced a stable POI model with a more severe phenotype and no spontaneous recovery. The ovarian tissue exhibited an immune imbalance characterized by an increased proportion of CD4^+^ T cells and a decreased proportion of Treg, which is consistent with the general characteristics of autoimmune diseases [21].

Elevated IFN-γ levels generally indicate the activation of Th1-type immune responses [22], whereas the upregulation of IL-10 may represent a regulatory immune feedback mechanism by which the host attempts to restrain excessive inflammation [23]. Imbalanced pro-inflammatory and anti-inflammatory signaling may directly govern the rate and severity of disease progression. In the present study, the more severe pathological phenotype observed in the EN-POI group is likely associated with Th1-dominant immune polarization and impaired Treg function, which compromises their capacity to effectively suppress effector T cell-mediated immune attacks against ovarian tissues. Notably, individual variations in immune responses among experimental mice still exist despite the stable overall pathological phenotype of the established model. Such inter-individual differences in immune activation intensity and cytokine secretion patterns may lead to slight heterogeneity in ovarian lesion severity across animals, which represents an inherent limitation of this autoimmune disease model.

Elevated levels of chemokines CCL4 and CCL5 specifically recruit immune cells such as monocytes into the ovarian microenvironment, thereby amplifying inflammatory responses and exacerbating tissue injury. Persistent inflammatory stimulation subsequently drives the initiation and progression of ovarian fibrosis [24]. Previous studies have demonstrated that activated T cells and monocyte/macrophage populations can secrete cytokines, including IFN-γ and TGF-β, to activate ovarian fibroblasts, resulting in excessive deposition of extracellular matrix [25,26]. Such fibrotic remodeling disrupts normal ovarian architecture and impairs follicular blood supply and nutritional support, thereby forming a vicious cycle: immune dysregulation → ovarian injury → aggravated fibrosis → further ovarian functional decline [27]. In the present study, the most prominent fibrotic phenotype in the EN-POI group, accompanied by severe T lymphocyte subset imbalance (elevated CD4^+^ T cells and reduced Tregs) and higher expression of pro-inflammatory factors (IFN-γ, CCL4, CCL5), is highly consistent with the aforementioned mechanistic cycle and our prior phenotypic results. These findings suggest that the severity of ovarian fibrosis may serve as a potential pathological indicator for evaluating the progression and prognosis of POI. Meanwhile, T cell dysregulation and dynamic alterations of associated cytokines may provide crucial immunological biomarkers for monitoring ovarian injury and fibrotic progression.

Proteomic analysis reveals that ovaries with POI exhibit molecular characteristics characterized by the coexistence of immune activation and metabolic suppression. Upregulated differentially expressed proteins are enriched in immune pathways such as RIG-I and IL-17, while downregulated proteins are associated with metabolic processes such as glycolysis and steroidogenesis. This finding links immune attack to functional failure of ovarian parenchymal cells: on the one hand, activated immune cells and the inflammatory factors they secrete can directly interfere with the normal metabolic and hormone synthesis functions of granulosa cells and theca cells [28]; on the other hand, the suppression of metabolic reprogramming may also weaken the follicles’ ability to respond to inflammatory stress, accelerating their atresia [29]. Combined with protein–protein interaction network analysis, this study identified six key hub proteins, including MMP9 and ISG15, and validated their association with disease progression using clinical data, further supporting their value as potential biomarkers or therapeutic targets. Naturally, the proteomics technology and analytical approaches employed in this study have their inherent limitations. First, mass spectrometry exhibits limited sensitivity for detecting proteins with extremely low abundance, highly hydrophobic proteins, or those with specific post-translational modifications, which may result in the failure to capture some important regulatory molecules. Second, the present study primarily focused on static protein expression levels, without in-depth investigation of dynamic regulatory characteristics such as post-translational modifications and subcellular localization. Consequently, it is challenging to fully elucidate the mechanisms underlying changes in protein functional activity. In the future, integrating multi-omics data (e.g., transcriptomics and metabolomics) for combined analysis will facilitate a more systematic illustration of the molecular network governing POI occurrence and progression, thereby refining and deepening the conclusions of this study.

Although the present study provides novel insights into the pathogenesis of immune-mediated POI, several inherent limitations should be objectively acknowledged. First, despite the identification of key hub proteins via proteomic profiling and their negative correlation with serum AMH levels in human POI cohorts, direct functional validation remains insufficient to define the causal roles of these candidate molecules in ovarian dysfunction. This is largely because the ovary is a complex glandular organ in which oocytes, granulosa cells, theca cells, and stromal cells interact through intricate paracrine and endocrine regulatory networks [30]. Conventional single-cell or co-culture systems are unable to recapitulate such multicellular crosstalk or mimic the dynamic in vivo immune microenvironment, thus precluding robust assessment of the specific effects of candidate proteins on follicular development and function [31]. Second, tissue-specific conditional transgenic mouse models represent the optimal approach for target functional validation; however, the generation of such models is extremely time-consuming and costly. Accordingly, in-depth functional validation will be addressed in our subsequent research.

With regard to sample size, all animal experiments in this study strictly adhered to the international 3R animal welfare principles (Replacement, Reduction, Refinement). We employed the minimum sufficient sample size that guaranteed adequate statistical power, which is a reasonable and standard practice for exploratory mechanistic studies.

Furthermore, although the hub proteins identified in this study provide potential molecular links to human POI pathogenesis, several critical translational limitations should be acknowledged. The pathophysiological processes, immune microenvironment, and disease progression in the pZP3-induced autoimmune mouse model are not fully identical to those of sporadic clinical POI in humans. Animal models inherently differ from human pathophysiology and cannot completely recapitulate the complex clinical heterogeneity, multifactorial etiology, and diverse onset patterns observed in human patients. Therefore, direct extrapolation of the present mechanistic findings and candidate molecular targets from mice to clinical applications remains preliminary. Collectively, the findings of this study should be regarded as exploratory and hypothesis-generating, laying a mechanistic basis for subsequent clinical translational research.

Moreover, the evidence from human samples in this study currently relies primarily on published public datasets with relatively incomplete information, which limits the strength of our inference regarding the “clinical predictive value” based on “correlation.” We aim to establish a larger human patient cohort to further validate the diagnostic value of these key proteins, thereby advancing the precision diagnosis and treatment of immune-mediated POI. In future translational research, efforts should be directed toward establishing a larger-scale, prospective cohort of human POI patients to further validate the levels of these protein biomarkers in serum or follicular fluid, as well as their associations with disease stage and activity. Simultaneously, organoids or more advanced co-culture systems should be utilized to partially simulate the ovarian microenvironment, enabling more comprehensive functional studies. These endeavors will ultimately promote the precise diagnosis and treatment of immune-mediated POI.

In summary, the present study first established a more clinically relevant, robust, and irreversible animal model of immune-mediated POI, addressing the limitation that most existing POI models rely on chemical-induced injury with insufficient exploration of immune pathogenesis. Through systematic investigations, we elucidated the central role of immune dysregulation—particularly aberrant T lymphocyte subsets and dysregulated pro-inflammatory cytokine profiles—in the onset and progression of POI, and further revealed the intrinsic association between T cell imbalance and ovarian fibrosis, thereby supplementing a novel mechanistic axis of immune dysregulation → ovarian injury → progressive fibrosis → ovarian functional decline. This study not only verifies that immune mediation is a crucial pathogenic pathway of POI and identifies the potential significance of core regulatory molecules, but also provides initial insights into immune-metabolic crosstalk in the diseased ovary, with the six identified hub proteins warranting further functional validation.

## 4. Materials and Methods

### 4.1. Mice

The mice used in the experiment were purchased from the Laboratory Animal Center of Nanjing Medical University, including 6-week-old female C57BL/6J mice (n = 30) and 10-week-old male C57BL/6J mice (n = 15), all of which were SPF grade. They were housed together in the biosecure animal laboratory at the Jiangning Campus of Nanjing Medical University. Housing conditions included appropriate temperature and humidity, a 12 h light/12 h dark cycle, and an ad libitum supply of food and water. The animal study protocol was approved by the Animal Ethics Committee of Nanjing Medical University (protocol code 1ACUC-2406035).

### 4.2. pZP3-Induced Autoimmune POI Model

We used peptidic zona pellucida 3 (pZP3) to induce autoimmune POI in a mouse model. Mice were randomly divided into three groups: control group, RE-POI group (conventional immunization), and EN-POI group (enhanced immunization), with 10 mice per group. First, pZP3 (amino acid sequence: NSSSSQFQIHGPR, 95% purity, TG-LG-16391, QYAOBIO, Shanghai, China) was dissolved in deionized water and mixed with an equal volume of complete Freund’s adjuvant (CFA) (HY-153808, MedChemExpress, Monmouth Junction, NJ, USA) or incomplete Freund’s adjuvant (FIA) (HY-153808A, MedChemExpress, USA) to form an emulsion. Subsequently, 0.15 mL of the pZP3-CFA emulsion was administered via multiple subcutaneous injections into the bilateral hind paw pads, abdominal, and dorsal neck regions of the mice. Two weeks later (i.e., on day 15), 0.15 mL of the pZP3-FIA emulsion was injected into the same regions. The EN-POI group received one additional round of injections compared to the RE-POI group. Meanwhile, the control group received an equal volume of PBS solution via the same injection method. Body weight was monitored weekly. Following model establishment, five female mice were randomly selected from each group for fertility assessment (detailed in Section 4.4). The remaining mice were euthanized within 2 weeks upon completion of the experiment. On the day of euthanasia, ovarian tissues were harvested for histological, functional, and proteomic analyses, while spleens were collected for flow cytometric analysis.

### 4.3. Vaginal Smears and Staining in Mice

Before the experiment and after the completion of immunization, the estrous cycle of mice (n = 10, per group) was continuously monitored for 8 consecutive days (approximately two complete cycles) in each period. At a fixed time each day (9:00–10:00 a.m.), a cotton swab moistened with sterile saline is gently inserted into the mouse’s vagina to obtain a smear of vaginal exfoliated cells. After staining, the cells are examined under a light microscope to assess their morphology. Based on previous studies [32,33], the stage of the estrous cycle (proestrus, estrus, diestrus, and anestrus) in mice was determined according to the relative proportions of nucleated epithelial cells, keratinized epithelial cells, and leukocytes.

### 4.4. Fertility Monitoring Experiment

Female mice with established POI models (Control, n = 5; RE-POI, n = 5; EN-POI, n = 5) were housed with wild-type C57BL/6J male mice known to have normal fertility in a 1:1 ratio. During cohabitation, vaginal plugs were examined at a fixed time every day to confirm whether mating had occurred. Female mice in which vaginal plugs were observed were housed individually, and their pregnancy status was continuously monitored; the date of parturition and the number of pups per litter were recorded. The fertility tracking experiment was conducted in three rounds.

### 4.5. H&E Staining and Follicle Counting

Ovarian samples were collected from different groups, fixed with 4% paraformaldehyde, and processed into paraffin-embedded specimens. These specimens were then cut into 5-micron-thick consecutive sections. Subsequently, hematoxylin–eosin (H&E) staining was performed according to the manufacturer’s instructions (HY-K0315, MedChemExpress, USA) to observe the morphological structure of the ovaries under an optical microscope. For each ovarian sample from individual mice, five randomly selected H&E-stained paraffin sections were examined under a microscope to observe the ovarian morphological structure. The number of follicles at each stage was counted and subjected to statistical analysis. To avoid double-counting the same follicle, only follicles with visible oocyte nuclei were counted. The classification of follicles at different stages was based on previous literature [34].

### 4.6. Masson Staining

Masson staining was performed using the Masson Stain Kit (60532ES58, Yeasen, Shanghai, China). Briefly, paraffin-embedded tissue sections were deparaffinized, and hematoxylin was applied for nuclear staining. After differentiation, the cytoplasm, muscle fibers, and erythrocytes were stained with acid fuchsin. Subsequently, sections were differentiated with phosphomolybdic acid solution, and collagen fibers were stained with aniline blue. Following sufficient rinsing with absolute ethanol, the sections were air-dried, mounted, and observed under an optical microscope.

### 4.7. Enzyme-Linked Immunosorbent Assay (ELISA)

Mice were anesthetized with tribromoethanol (DW3101, Dowobio, Shanghai, China), and blood samples were collected from the retroorbital venous plexus. Serum was separated by centrifugation for hormone detection in accordance with standard ELISA protocols. Absorbance (OD values) was measured at 450 nm using a microplate reader, and sample concentrations were calculated accordingly. The ELISA kits used were as follows: FSH (RK04237, ABclonal, Shanghai, China), AMH (RK09261, ABclonal, China), and E2 (RK00651, ABclonal, China).

### 4.8. Multi-Parameter Flow Cytometric Co-Analysis Technology

Mice were anesthetized with isoflurane, and blood samples were collected from the retroorbital venous plexus. Serum was isolated via centrifugation after collection. Multiplex cytokine detection was carried out using a commercial bead-based assay kit (ABclonal, Cat. No. RK05203, China) following the general procedure provided by the manufacturer. Antigen-specific antibody-coated microspheres were incubated with serum samples to form immune complexes. After washing steps, signal detection was performed on an ABplex-100 multi-parameter flow cytometer (ABclonal, China). Fluorescent signals from microsphere identifiers and bound reporter molecules were determined via dual-laser scanning for qualitative identification and quantitative analysis, respectively.

### 4.9. Flow Cytometry

Mouse spleens were thoroughly minced and filtered through a 70-μm mesh to obtain single-cell suspensions. Prior to analysis, dead cells were eliminated using FVD (Cat. No. 65-0865-14, eBioscience, San Diego, CA, USA). For cell-surface staining, 1 × 10^6^ cells per sample were incubated with the corresponding antibodies in staining buffer (PBS + 2.5% fetal bovine serum) for 15 min at room temperature in the dark. For nuclear staining, cells were fixed and permeabilized at 4 °C for 1 h using fixation/permeabilization buffer (Cat. No. 00-5523-00, eBioscience, USA). For intracellular cytokine staining, cells were stimulated with cell activation mix (Cat. No. 423304, BioLegend, San Diego, CA, USA) at 37 °C for 4 h, followed by fixation and permeabilization with buffer (Cat. No. AB2869008, BD Biosciences, Milpitas, CA, USA) at 4 °C for 20 min according to the manufacturer’s protocol. Stained cells were analyzed on a FACSCanto II flow cytometer (BD Biosciences), and data were processed using FlowJo software (V 10.6.1, BD Biosciences, USA). The specific antibodies used are detailed in the table below.
**Name****Brand****Item Number**CD45eBioscience35-0451-82CD4eBioscience48-0041-82CD8aeBioscience69-0081-82NK1.1eBioscience406-5941-82CD19eBioscience17-0193-82Ly6ceBioscience25-5932-80

### 4.10. Tissue Lysis and Homogenization

Intact ovarian tissues from Control (n = 3) and EN-POI (n = 3) mice were subjected to quantitative proteomics analysis at Shanghai OE Biotech Co., Ltd. (Shanghai, China). Samples were lysed with RIPA lysis buffer (P0013B, Beyotime Biotechnology, Shanghai, China) supplemented with phosphatase inhibitor cocktail (P1082, Beyotime Biotechnology, China) and 1 mM PMSF (ST507-10 mL, Beyotime Biotechnology, China). The mixture was transferred into 2.0 mL centrifuge tubes and homogenized twice at −35 °C and 60 Hz for 120 s using a cryogenic grinder. Tissue homogenates were centrifuged at 12,000 rpm for 10 min at 4 °C, and the supernatant was collected and re-centrifuged under the same conditions. Protein concentration was quantified using the Bicinchoninic Acid (BCA) assay (23225, Thermo Fisher Scientific, Waltham, MA, USA).

### 4.11. SDS-PAGE Gel Electrophoresis

30 μg protein per sample was separated by 4–12% SDS-PAGE. The gel was stained with Coomassie Brilliant Blue using an eStain LG protein staining instrument and imaged with a fully automatic digital gel image analysis system. The SDS-PAGE profiles of all biological replicates are provided in Figure S6 of the Supplementary Materials for the verification of protein integrity and sample loading consistency.

### 4.12. Protein Digestion and Desalting

50 μg protein lysate per sample was normalized to equal concentration and volume using RIPA lysis buffer. Pretreated SP3 magnetic beads and an appropriate volume of 100% acetonitrile (ACN) were added to the protein solution, followed by incubation at room temperature (RT) for 20 min. After brief centrifugation, the supernatant was discarded, and the beads were washed twice with 70% ethanol and twice with 70% ACN sequentially. The beads were resuspended in 50 mM ammonium bicarbonate (ABC) solution, supplemented with 25 mM dithiothreitol (DTT), and incubated at 55 °C for 30 min. Chloroacetamide (CAA), trypsin, and LysC were then added, and proteolysis was performed at 37 °C with shaking at 1500 rpm.

After digestion, peptides were desalted using a SOLA™ SPE 96-well plate. The plate was activated and equilibrated three times (repeated twice) with 200 μL methanol and 200 μL 0.1% formic acid in water, respectively. 200 μL peptide samples were loaded under vacuum at ~1 mL/min, and loading was repeated once. The plate was washed three times with 200 μL of 0.1% formic acid aqueous solution, and peptides were eluted three times with 150 μL of 50% ACN-water (0.1% FA), yielding 450 μL eluate, which was vacuum-dried.

### 4.13. Mass Spectrometric Analysis

High-resolution LC-MS/MS analysis was performed using a Vanquish neo liquid chromatography system (Thermo Fisher Scientific, USA) coupled to a timsTOF HT mass spectrometer (Bruker, Bremen, Germany). Prior to injection, each sample was spiked with the iRT internal standard at a 1:20 volume ratio. iRT standards (Biognosys, Thermo Fisher Scientific, USA) were used for chromatographic calibration and quantitative quality control. Relative protein quantification in ovarian tissues was achieved by comparing peptide signal intensities between the experimental and control groups. See Table 1 and Table 2 below for the detailed chromatographic and mass spectrometry (MS) conditions.

### 4.14. DIA Data Processing and Statistical Analysis

Raw MS data were processed using DIA-NN v2.2.0 for database searching, peptide matching, and protein quantification to screen differentially expressed proteins (DEPs) across mouse ovarian tissues. Raw quantitative data were log-transformed, and intergroup differences were assessed by a dual-criterion strategy combining fold change (FC) and Student’s *t*-test *p*-values. Log2(fold change) was defined as the ratio of mean protein abundance in the experimental group to that in the control group. DEPs were screened according to the thresholds: *p* < 0.05, FC ≥ 1.5 or FC ≤ 1/1.5. This workflow identified qualified DEPs for subsequent mechanistic research. See Table 3 below for the DIA-NN software analysis parameter settings.

### 4.15. Statistical Analysis

Prior to parametric analysis, the Shapiro–Wilk test (α = 0.10) was used to assess the normality of all data. Homogeneity of variances was verified using the Levene test (α = 0.05). To address the potential inflation of Type I errors caused by multiple hypothesis testing, the Bonferroni method was applied to the analysis of results involving correlated variables. Normally distributed data were compared using two-tailed Student’s t-tests or one-way analysis of variance (ANOVA). When data were not normally distributed, two-tailed Mann–Whitney U tests and two-tailed Kruskal–Wallis tests were employed. Results were considered statistically significant when *p* < 0.05. Pearson correlation analysis was used to determine correlations between gene expression levels. Evaluation parameters within groups are presented as the mean and standard error of the mean (SEM) for each group. Unless otherwise specified, each experiment was repeated at least three times. Statistical analysis was performed using GraphPad Prism 10.0 software.

## 5. Conclusions

By optimizing the establishment of the pZP3-induced autoimmune POI model, we characterized immune pathological changes featured by CD4^+^ T cell and Treg imbalance during ovarian injury. On this basis, using unbiased DIA proteomic sequencing, we identified key differentially expressed proteins and further pinpointed six core hub proteins: Mmp9, Isg15, Ikbke, Siglec1, Pf4, and Cdkn1b. This study for the first time constructed the molecular regulatory network underlying immune-mediated ovarian insufficiency, revealing that these hub proteins closely link immune dysregulation, ovarian metabolic disturbance, and progressive tissue fibrosis. These findings expand the current molecular mechanism landscape of immune-driven POI, provide previously unrecognized molecular targets, and lay a solid foundation for subsequent functional validation and the development of targeted intervention strategies.

## Figures and Tables

**Table 1:** Chromatographic conditions.

Time (min)	Gradient	Flow Rate (μL/min)
0	7.5% B	0.7
8	35% B	0.7
9	100% B	0.7
10	100% B	0.7

**Table 2:** MS conditions.

Items	Para.
Capillary	1.6 KV
Dry Temperature	180 °C
Dry Gas	3.2 L/min
Mass Range	300–1500 *m*/*z*
Ion Mobility	0.7–1.3
Collision Energy	20–59 eV
Ramp Time	50 ms

**Table 3:** Parameters for DIA-NN data processing.

Item	Value
Enzyme	Trypsin
Max Missed Cleavages	2
Fixed Modifications	Carbamidomethyl (C)
Variable Modifications	Oxidation (M), Acetyl (Protein N-term)
Database Pattern	Target-Reverse
PSM (Peptide-Spectral Matching) FDR	0.01
Protein FDR	0.01

## Data Availability

The mass spectrometry proteomics data have been deposited to the ProteomeXchange Consortium via the iProX partner repository (https://proteomecentral.proteomexchange.org) with the dataset identifier PXD077415. The link to access these data is provided in the Data Availability Statement section at the end of the article: https://www.iprox.cn/page/project.html?id=IPX0016770000 (accessed on 29 April 2026). Further inquiries can be directed to the corresponding author.

## Supplementary Materials

The following supporting information can be downloaded at: https://www.mdpi.com/article/10.3390/ijms27104270/s1.

## Abbreviations

The following abbreviations are used in this manuscript:

POI	premature ovarian insufficiency
pZP3	polypeptide Zone Pellucida 3
FSH	follicle-stimulating hormone
AMH	anti-Müllerian hormone
E2	estradiol
ELISA	enzyme-linked immunosorbent assays
PPI	protein–protein interaction
LC-MS	liquid chromatography–mass spectrometry
PCA	principal component analysis
DEPs	differentially expressed proteins
BP	biological processes
CC	cellular components
MF	molecular functions
IFN-γ	interferon-γ
