# Supplementary material for: Establishment of an Autoimmune Premature Ovarian Insufficiency Mouse Model with Proteomic Analyses: An Exploratory Study
Source: Int J Mol Sci. 2026 May 11;27(10):4270. doi: 10.3390/ijms27104270 (PMC13207734; doi:10.3390/ijms27104270)
Supplement: Supplementary file 1 [file ijms-27-04270-s001.zip › ijms-4251290-supplementary.pdf]

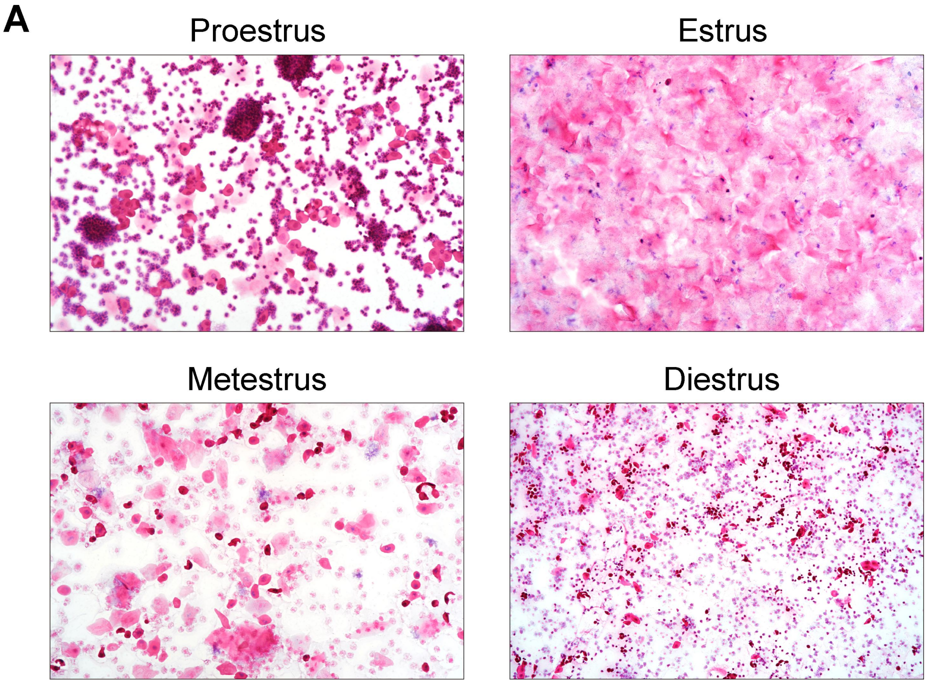

Figure S1. Typical vaginal smear morphology showing the four phases of the estrous cycle: proestrus (P), estrus (E), metestrus (M), and diestrus (D).

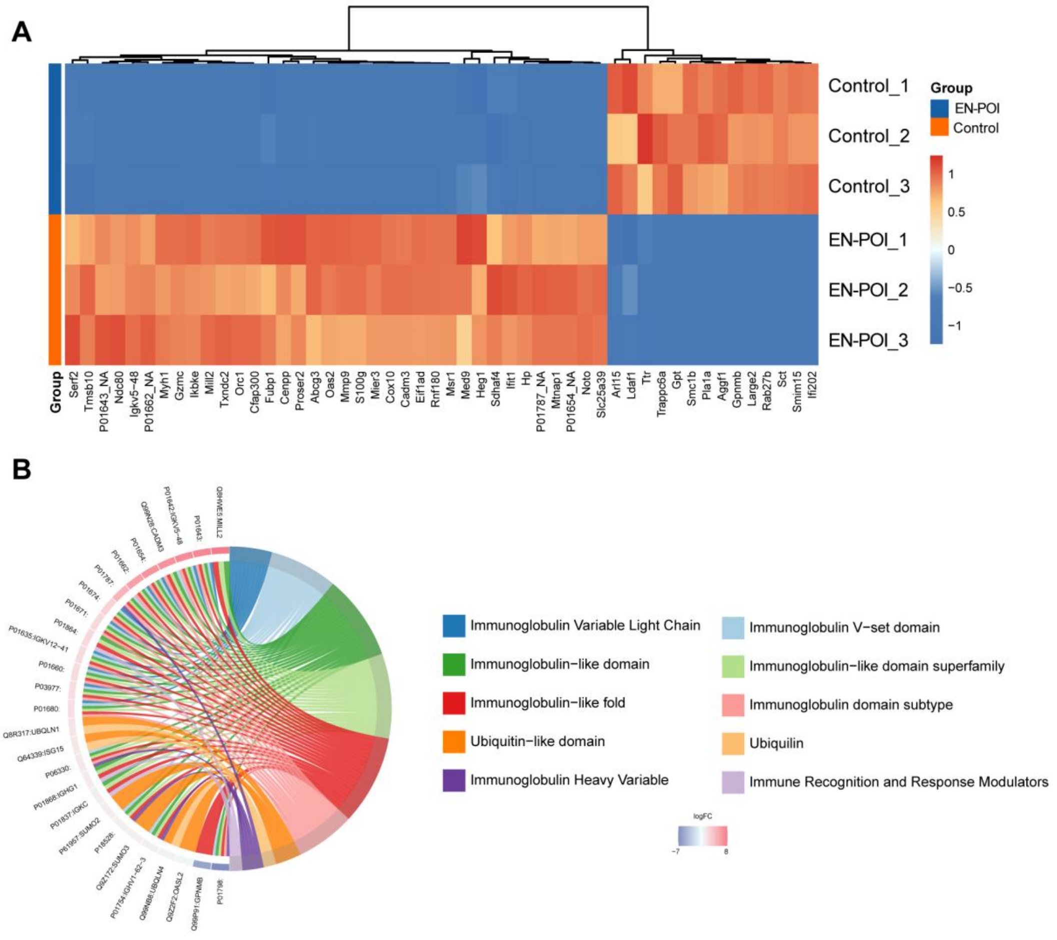

Figure S2.Proteomic sequencing and functional enrichment analysis of ovaries from POI and control mice. (A) Volcano plot of differentially expressed genes (DEPs) between the POI and control groups. (G) Circular plot of protein domain enrichment for DEPs.

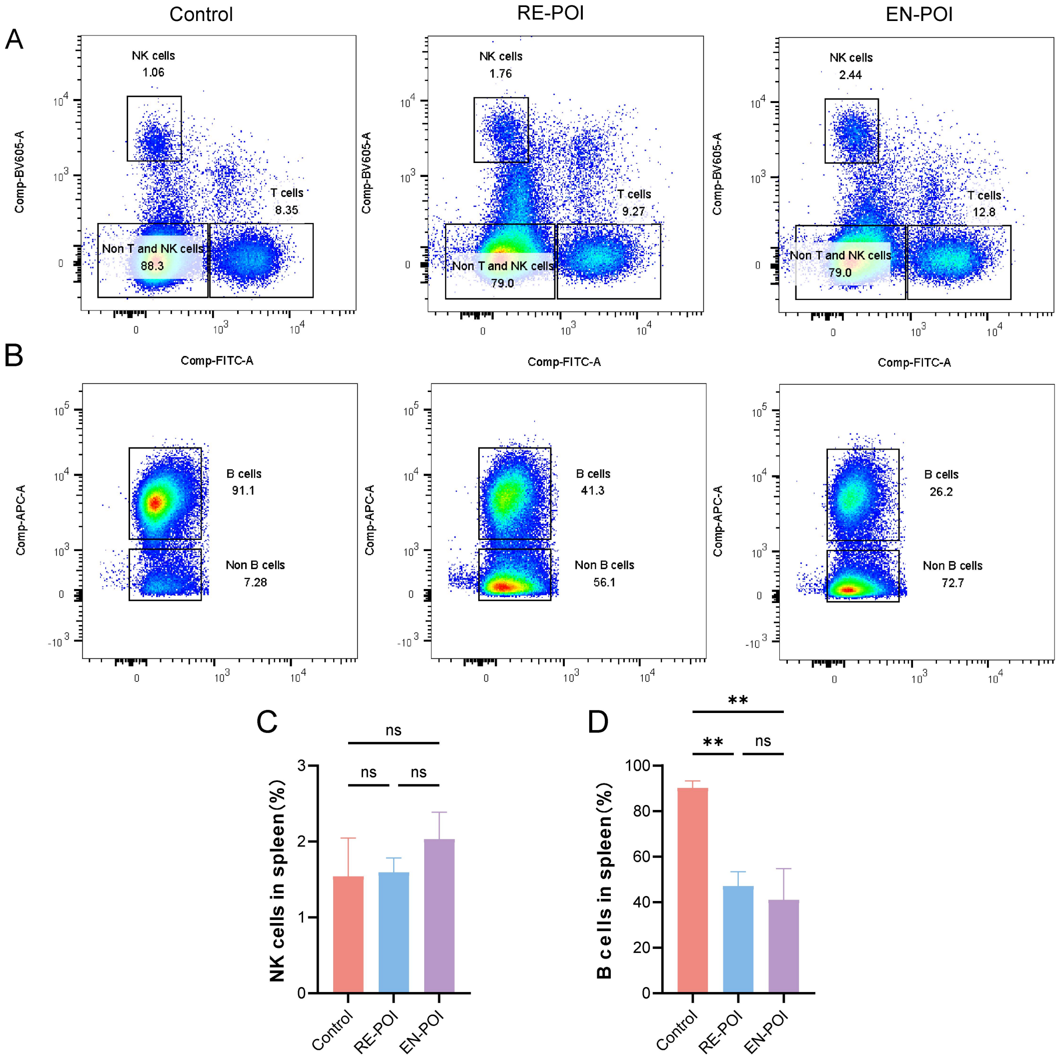

Figure S3: Immune cell subsets in the mouse spleen. (A) Flow cytometric analysis of the proportion of NK cells in the spleen of POI mice. (B) Flow cytometric analysis of the proportion of B lymphocytes in the spleen of POI mice. (C) Statistical analysis of the proportion of NK cells in the spleen of POI mice. (D) Statistical analysis of B lymphocytes in the spleen of POI mice. *p<0.05; ***p<0.001; ns, no statistical difference.

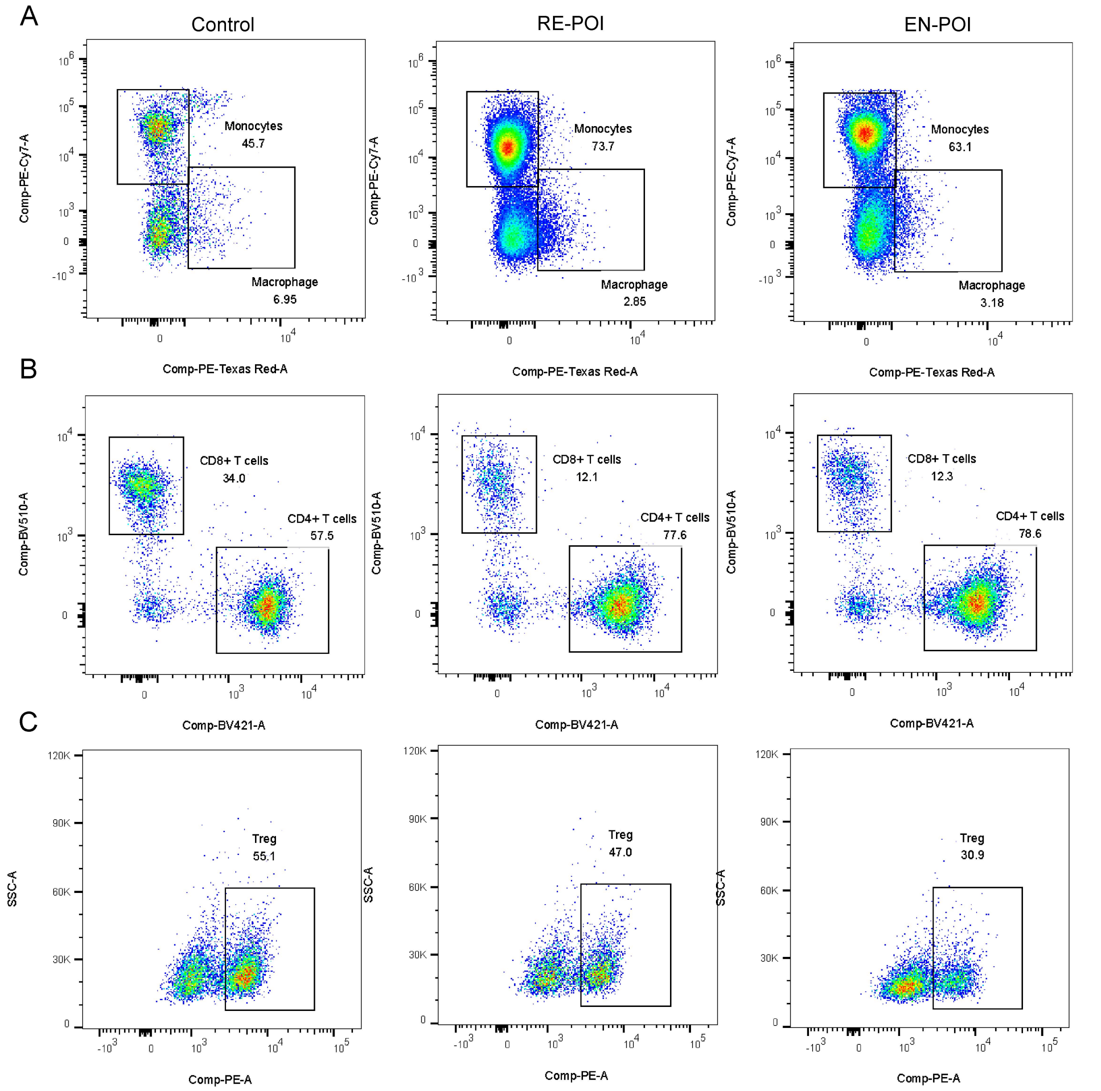

Figure S4. Immune cell subsets in the mouse spleen. (A) Flow cytometric analysis of the proportion of mononuclear phagocytes in the spleen of POI mice. (B) Flow cytometric analysis of the proportion of T lymphocytes in the spleen of POI mice. (C) Flow cytometric analysis of the proportion of Treg cells in the spleen of POI mice.

A

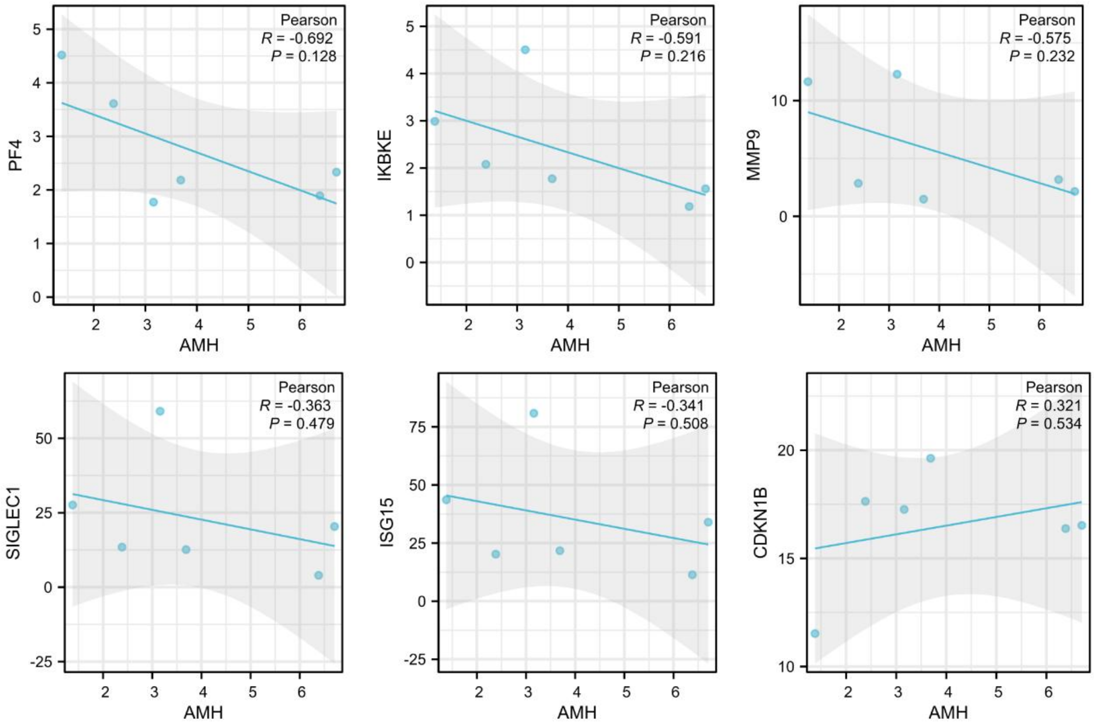

Figure S5: Analysis of the correlation between the Pf4, Ikbke, Mmp9, Siglec1, Isg15, and CDKN1B genes and the ovarian reserve marker AMH

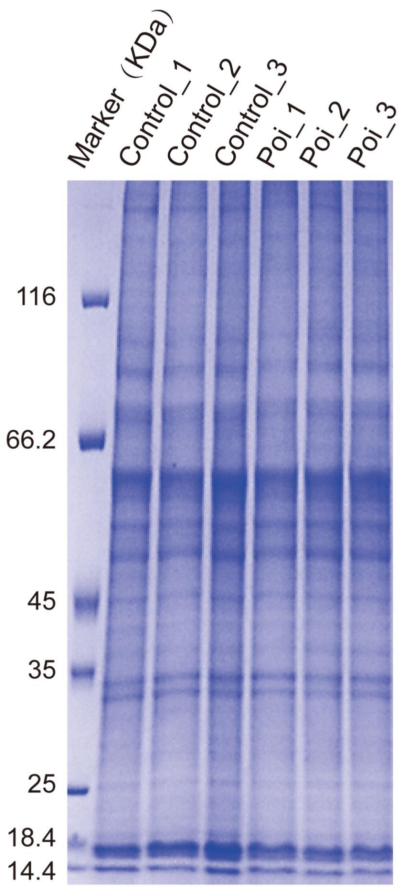

Figure S6: SDS-PAGE electrophoresis profiles of ovarian tissue protein samples from all biological replicates
